# Characterization of proximal pulmonary arterial cells from chronic thromboembolic pulmonary hypertension patients

**DOI:** 10.1186/1465-9921-13-27

**Published:** 2012-03-27

**Authors:** Rozenn Quarck, Marijke Wynants, Alicja Ronisz, Maria Rosario Sepulveda, Frank Wuytack, Dirk Van Raemdonck, Bart Meyns, Marion Delcroix

**Affiliations:** 1 Center for Pulmonary Vascular Diseases, Respiratory Disease Department, Katholieke Universiteit and Universitaire Ziekenhuizen Leuven, Leuven, Belgium; 2 Molecular Cell Biology Department, Katholieke Universiteit, Leuven, Belgium; 3 Thoracic Surgery and Cardiac Surgery Department, Universitaire Ziekenhuizen Leuven, Leuven, Belgium; 4 Cardiac Surgery Department, Universitaire Ziekenhuizen Leuven, Leuven, Belgium; 5 Department of Pneumology; Universitaire Ziekenhuizen Leuven, Herestraat 49, B-3000 Leuven, Belgium

**Keywords:** Chronic Thromboembolic Pulmonary Hypertension, Smooth Muscle Cells, Endothelial cells, Vascular Remodeling

## Abstract

**Background:**

Chronic thromboembolic pulmonary hypertension (CTEPH) is associated with proximal pulmonary artery obstruction and vascular remodeling. We hypothesized that pulmonary arterial smooth muscle (PASMC) and endothelial cells (PAEC) may actively contribute to remodeling of the proximal pulmonary vascular wall in CTEPH. Our present objective was to characterize PASMC and PAEC from large arteries of CTEPH patients and investigate their potential involvement in vascular remodeling.

**Methods:**

Primary cultures of proximal PAEC and PASMC from patients with CTEPH, with non-thromboembolic pulmonary hypertension (PH) and lung donors have been established. PAEC and PASMC have been characterized by immunofluorescence using specific markers. Expression of smooth muscle specific markers within the pulmonary vascular wall has been studied by immunofluorescence and Western blotting. Mitogenic activity and migratory capacity of PASMC and PAEC have been investigated *in vitro*.

**Results:**

PAEC express CD31 on their surface, von Willebrand factor in Weibel-Palade bodies and take up acetylated LDL. PASMC express various differentiation markers including α-smooth muscle actin (α-SMA), desmin and smooth muscle myosin heavy chain (SMMHC). In vascular tissue from CTEPH and non-thromboembolic PH patients, expression of α-SMA and desmin is down-regulated compared to lung donors; desmin expression is also down-regulated in vascular tissue from CTEPH compared to non-thromboembolic PH patients. A low proportion of α-SMA positive cells express desmin and SMMHC in the neointima of proximal pulmonary arteries from CTEPH patients. Serum-induced mitogenic activity of PAEC and PASMC, as well as migratory capacity of PASMC, were increased in CTEPH only.

**Conclusions:**

Modified proliferative and/or migratory responses of PASMC and PAEC *in vitro*, associated to a proliferative phenotype of PASMC suggest that PASMC and PAEC could contribute to proximal vascular remodeling in CTEPH.

## Background

Chronic thromboembolic pulmonary hypertension (CTEPH) is one of the main causes of pulmonary hypertension. CTEPH is characterized by the presence of unresolved thromboemboli associated to fibrous stenosis in the proximal pulmonary arteries. This is resulting in obstruction of proximal pulmonary arteries, increased pulmonary vascular resistance, pulmonary hypertension and progressive right heart failure. It might be caused by single or recurrent pulmonary embolism and/or local formation of thrombi. Proximal obliteration of pulmonary artery, removable by pulmonary endarterectomy (PEA), is the major feature observed in CTEPH [1].

The mechanisms responsible for permanent vessel obstruction remain poorly understood. Proximal lesions share similarities with atherosclerotic plaques, including media thickening and neointima formation [2]. Dysregulation of thrombosis and/or thrombolysis has been described [3-5] as well as an elevated prevalence of inflammatory diseases [6] and mild systemic inflammation [7]. Although vascular remodeling and increased cellularity have been observed in the proximal vascular lesions [8], cellular protagonists have not been identified and respective involvement of both endothelial (PAEC) and smooth muscle cells (PASMC) remains unexplored.

CD31 or PECAM-1 (platelet endothelial cell adhesion molecule-1), a member of the immunoglobulin superfamily, is a transmembrane glycoprotein homogeneously expressed by all human pulmonary EC [9]. von Willebrand Factor (vWF), a glycoprotein mediating platelet adhesion to subendothelium, is mainly produced by endothelial cells where it is stored in the Weibel-Palade bodies [9]. The scavenger receptor expressed by endothelial cells can bind fluorescent DiI-labeled acetylated-LDL (ac-LDL) and is currently used to characterize EC [10]. α-smooth muscle actin (α-SMA), desmin and smooth muscle myosin heavy chain (SMMHC) are constitutive proteins of the cytoskeleton involved in the smooth muscle contractile functions and are differentially expressed during smooth muscle differentiation [11].

Enhanced thrombosis appeared not to be the major cause of permanent obstruction of proximal pulmonary arteries. We consequently hypothesized that both PAEC and PASMC derived from the pulmonary arterial wall of CTEPH patients may be active contributors to the physiopathology of CTEPH. Our current objectives were to i) isolate EC and SMC from proximal and (sub)segmental pulmonary arterial wall of CTEPH patients, ii) further characterize them using specifics markers and iii) investigate their proliferating and migrating capacities *in vitro*.

## Methods

### Study population

Proximal pulmonary vascular material was obtained from i) 16 CTEPH patients, ii) 12 patients with non-thromboembolic pulmonary hypertension (PH) (4 with idiopathic pulmonary arterial hypertension (PAH), 1 with PAH associated to congenital heart disease and 7 with PH due to lung diseases) and 15 lung donors. Hemodynamic parameter evaluation was performed by right heart catheterization at the time of lung transplantation or PEA. The study protocol was approved by the Institutional Ethics Committee of the University Hospital of Leuven and participants gave written informed consent.

### Tissue collection

**Figure 1:**
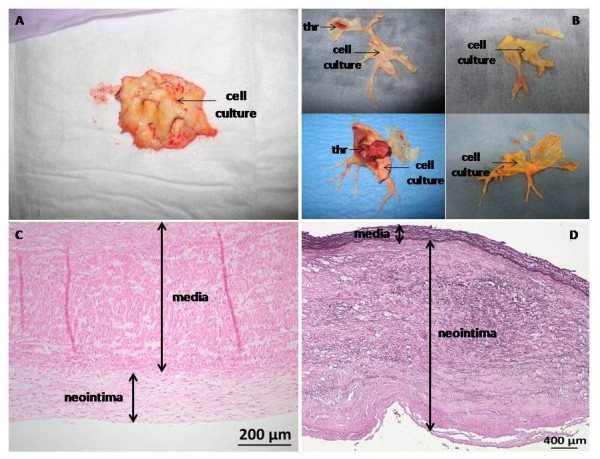
**Pulmonary vascular material collected during lung transplantation from a patient with PAH (A) and stained by hematoxylin and eosin (C) and during PEA from patients with CTEPH (B) and stained for elastin (D)**. The presence of a thrombus (THR) was mentioned and the piece of tissue further used to isolate PAEC and PASMC (cell culture) was also indicated.

At the time of lung transplantation or PEA, a 2-cm piece of proximal pulmonary artery was collected (Figure 1A &1B). The piece used to isolate cells was free of any thrombotic material and comprised media (only a part regarding PEA samples) and neointima (Figure 1). A piece of segmental or sub-segmental pulmonary artery (diameter 5 to 10 mm) was also collected at the time of PEA.

### Neointima thickness

A contiguous piece of artery was fixed in 4% paraformaldehyde, paraffin-embedded and 7-μm sections were performed. Sections were deparaffinized and rehydrated. Nuclei and cytoplasm were stained by hematoxylin and eosin, respectively. Staining of elastin was performed using the Verhoeff's van Gieson method. Neointima thickness was measured as the maximal distance from the lumen to the internal elastic lamina.

### Isolation of PAEC and PASMC

Proximal PAEC were obtained by collagenase digestion followed by immunomagnetic separation using anti-CD31 monoclonal antibody-labeled beads (Miltenyi Biotec, Utrecht, The Netherlands). Proximal PASMC were isolated using an explant-outgrowth method [12]. All experiments were carried out with cells, which have undergone less than 7 passages.

### Cell culture media

EC were cultured in M199 medium (Life Technologies, Gent, Belgium), supplemented with 20% fetal bovine serum (FBS), 100 U.mL^-1^ penicillin, 100 μg.mL^-1 ^streptomycin, 1.25 μg.mL^-1 ^fungizone (Life Technologies), 10 U.mL^-1 ^heparin (Aventis, Brussels, Belgium) and 5 ng.mL^-1 ^α-FGF (R&D Systems, Abington, UK). SMC were grown in DMEM medium (Life Technologies) supplemented with 10% FBS, penicillin, streptomycin and fungizone.

### Cell characterization and immunofluorescence on pulmonary arterial tissue

PAEC phenotype was characterized by labeling cells with diI-Ac-LDL (Tebu, Le Perray en Yvelines, France) and by immunofluorescence using antibodies against CD31 and against vWF (Dako, Heverlee, Belgium). PASMC phenotype was characterized by immunofluorescence using antibodies against α-SMA, desmin (Dako) and human SMMHC (Biomedical Technologies, Stoughton, MA). Localization of α-SMA, desmin and SMMHC has been performed on 10-μm tissue cryosections by immunofluorescence.

### Western blotting

Identification of α-SMA and desmin in pulmonary vascular tissue was performed by Western blotting using specific primary antibodies against α-SMA or desmin. β-actin (Abcam, Cambridge, UK) was used as an internal control. Horseradish peroxidase-conjugated donkey anti-rabbit IgG for anti-desmin and anti-mouse IgG (Jackson, Suffolk, UK) for anti-αSMA and anti-β-actin were used as secondary antibodies. Peroxidase staining was revealed with a chemiluminescence kit (GE Healthcare, Buckinghamshire, UK) and performed with films exposed at room temperature. Protein expression was quantified using the Photoprint imaging system and the BIO-1D software (Vilber Lourmat, Marne-la-Vallée, France).

### Proliferation of PASMC and PAEC

Subconfluent PASMC and PAEC were starved for 24 h in medium supplemented with 0.2% FBS. Mitogenic activity of PAEC and PASMC was measured in the presence of 5% and 10% FBS, respectively plus 0.5 μCi.mL^-1 ^of [^3^H]-thymidine (74 GBq.mmol^-1^; GE Healthcare) for 48 h. Radioactivity incorporation was quantified as previously described [13]. [^3^H]-thymidine incorporation assays have been validated by cell counting for both PASMC and PAEC.

### Migration of PASMC

Subconfluent PASMC were starved for 24 h in DMEM supplemented with 0.2% FBS. PASMC migration was evaluated using a scratch wound assay in the presence of 10% FBS for 36 hours, as previously described [12].

### Statistical analysis

Database management and statistical analyses were performed using SAS Enterprise Guide 4.1 (SAS Inc., Cary, North Carolina) and GraphPad Prism 4.01 (GraphPad Software Inc., La Jolla, California). Data were expressed as mean ± SD. Differences between the 2 or 3 groups were analyzed using Student t-test or two-way ANOVA test followed by post-hoc tests. A value of p ≤ 0.05 was considered statistically significant. All p values were for 2-sided tests.

The detailed "Method" section is available in the Additional file 1.

## Results

The patient characteristics, from which pulmonary vascular material has been collected, are displayed in the Table 1. A table containing individual patient characteristics is available in the Additional file 2.

**Table 1:** Patient characteristics

	*Non-thromboembolic PH, n = 12*	*CTEPH, n = 16*
Age	50 ± 9	59 ± 13
Gender, female, %	23	81
mPAP, mm Hg	41 ± 9	42 ± 12
RAP, mm Hg	13 ± 4	11 ± 5
TPR, dyne.sec.cm^-5^	681 ± 469	1206 ± 574
CO, L.min^-1^	5.8 ± 1.9	3.1 ± 1.0

mPAP, mean pulmonary arterial pressure; RAP, right atrial pressure; TPR, total pulmonary vascular resistance; CO, cardiac output. Hemodynamic parameters have been measured at the time of PEA or lung transplantation.

Mean pulmonary arterial pressure (mPAP) was similar in CTEPH and in non-thromboembolic PH patients, whereas CTEPH patients displayed a significantly higher total pulmonary resistance (p = 0.04) and lower cardiac output (p < 0.0001) compared to non-thromboembolic PH patients. The pulmonary vascular material collected consisted in neointima and media (Figure 1C and 1D). However, neointima thickness strongly differed between CTEPH and non-thromboembolic patients with median (range) of 1.54 mm (0.46-2.08) *vs*. 0.26 mm (0.04-1.34), respectively (Mann-Withney, p < 0.0001).

### PAEC and PASMC characterization

**Figure 2:**
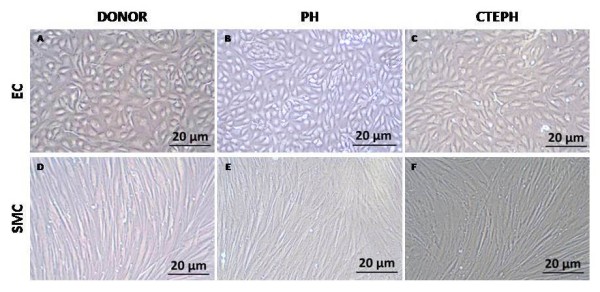
**Proximal PAEC and PASMC**. Subconfluent PAEC **(A, B, C) **and PASMC **(D, E, F) **isolated from proximal pulmonary arteries of lung donors (A, D), non-thrombembolic PH patients **(B, C) **and from CTEPH patients **(C, F)**.

Subconfluent proximal PAEC isolated from proximal pulmonary arteries of lung donors, patients with non-thromboembolic PH and CTEPH patients harbor typical "cobblestone" morphology, whereas PASMC display a "hills and valleys" organization (Figure 2).

**Figure 3:**
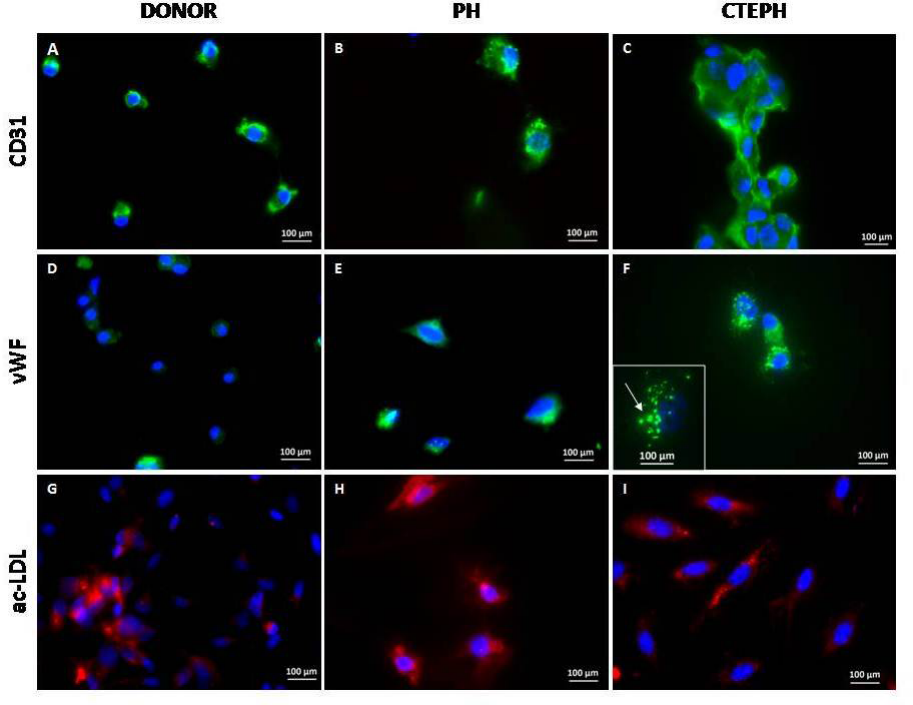
**PAEC characterization**. Proximal PAEC derived from lung donors (A, D, G), non-thromboembolic PH patients **(B, E, H) **and CTEPH patients **(C, F, I) **were immuno-labeled with antibodies raised against CD31 **(A, B, C) **and vWF **(D, E, F) **and stained with Dil-Ac-LDL **(G, H, I)**. Nuclei were counterstained using DAPI (blue). Insert **F**, the arrow indicates the presence of Weibel-Palade bodies where vWF is stored.

PAEC derived from lung donors, non-thromboembolic PH and CTEPH patients expressed CD31 at their surface, contained vWF in Weibel-Palade bodies and are able to take up acetylated LDL (Figure 3).

**Figure 4:**
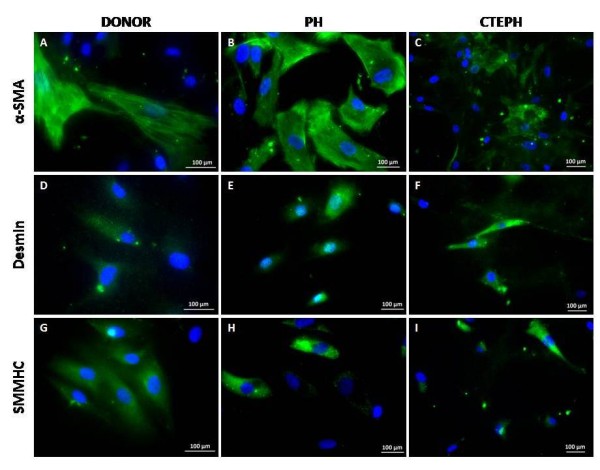
**PASMC characterization**. Proximal PASMC derived from lung donors **(A, D, G)**, non-thromboembolic PH patients **(B, E, H) **and CTEPH patients **(C, F, I) **were stained with antibodies raised against α-SMA **(A, B, C)**, desmin **(D, E, F) **and SMMHC **(G, H, I)**. Nuclei were counterstained using DAPI (blue).

PASMC populations derived from lung donors, non-thromboembolic PH and CTEPH patients displayed α-SMA filaments and expressed desmin and SMMHC (Figure 4).

### Differentiation marker expression and distribution in pulmonary arterial tissue

**Figure 5:**
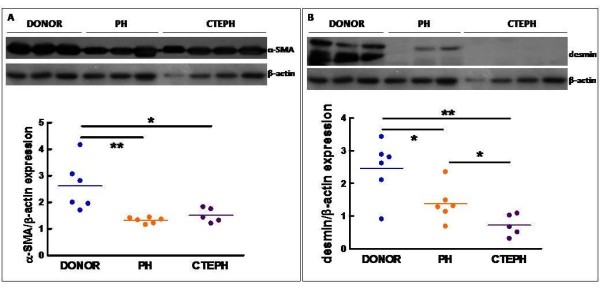
**Expression of SMC differentiation markers in pulmonary arteries**. Frozen pieces of main pulmonary artery were homogenized, lysed, 100 μg of protein samples were submitted to SDS-PAGE and electroblotted. Immunodetection of α-SMA **(A)**, desmin **(B) **and β-actin **(A, B) **was carried out by Western blotting using specific antibodies. Protein molecular mass was estimated in kDa using standard markers. Protein expression was quantified by Western blotting densitometry. Results are expressed as the ratio of the band volume of α-SMA or desmin to that of β-actin. Donors (n = 6), CTEPH (n = 5) and PH (n = 6). PH group: circle, PAH; triangle, non PAH-PH. **(A) **ANOVA, p = 0.003; *p < 0.05; **p < 0.01. **(B) **ANOVA, p = 0.002; *p < 0.05; **p < 0.01.

**Figure 6:**
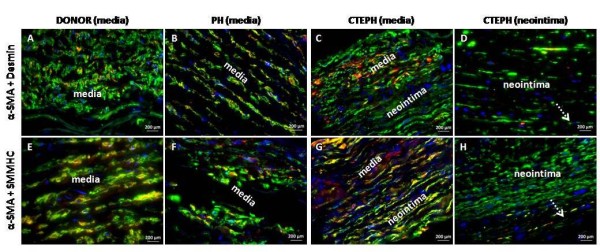
**Desmin, SMMHC and α-SMA distribution and co-localization in pulmonary arteries**. Sections of proximal pulmonary arteries from lung donors (A, E), non-thromboembolic PH patients **(B, F)**, CTEPH patients **(C, D, G, H) **were stained with antibodies raised against desmin and α-SMA **(A, B, C, D) **or against SMMHC and α-SMA **(E, F, G, H)**. Nuclei were counterstained using DAPI (blue).

The expression of α-SMA was 50% and 42% significantly lower in pulmonary artery tissue of non-thromboembolic PH and CTEPH patients, respectively, compared to lung donors (Figure 5A). Similarly, expression of desmin was 44% and 71% lower in pulmonary artery tissue of non-thromboembolic PH and CTEPH patients, respectively compared to lung donors (Figure 5B). Most α-SMA positive cells in the media from lung donors, non-thromboembolic PH and CTEPH patients express desmin (Figure 6A, B, C) and SMMHC (Figure 6E, F, G), whereas only a low proportion of SMC in the neointima from CTEPH patients were co-stained for α-SMA and desmin (Figure 6D) or α-SMA and SMMHC (Figure 6H).

### PAEC and PASMC proliferation and migration

**Figure 7:**
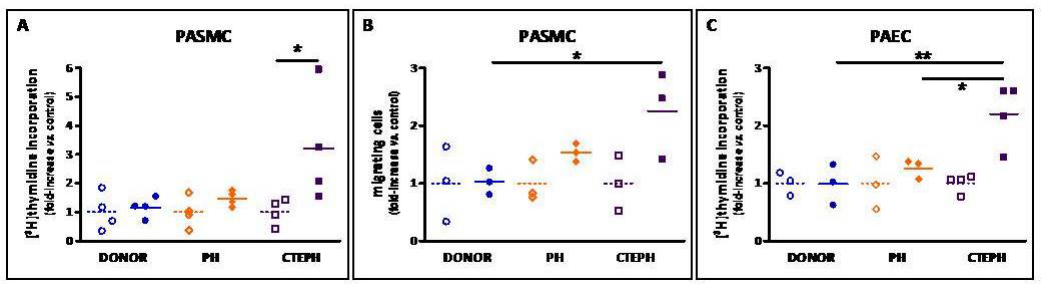
**Proximal pulmonary vascular cell proliferating and migrating capacity**. **(A) **Mitogenic activity of proximal PASMC isolated from lung donors (n = 4), non-thromboembolic PH (n = 4) and CTEPH patients (n = 4). Subconfluent cells were starved for 24 h in 0.2% FBS medium. Mitogenic activity was measured in the presence of 0.2% (control) or 10% FBS plus 0.5 μCi.mL^-1 ^of [^3^H]-thymidine for 36 h. ANOVA, p = 0.02; *p < 0.05. **(B) **Migrating capacity of proximal PASMC isolated from lung donors (n = 3), non-thromboembolic PH (n = 3) and CTEPH patients (n = 3). Subconfluent cells were starved for 24 h in 0.2% FBS medium. PASMC migration was evaluated using a scratch wound assay in the presence of 0.2% (control) or 10% FBS for 36 hours. ANOVA, p = 0.02; *p < 0.02. **(C) **Mitogenic activity of proximal PAEC isolated from lung donor (n = 1), non-thromboembolic PH (n = 3) and CTEPH patients (n = 4). Subconfluent cells were starved for 24 h in 0.2% FBS medium. Mitogenic activity was measured in the presence of 0.2% (control) or 5% FBS as described above. ANOVA, p = 0.0002, *p < 0.02, **p < 0.002. *Open symbols*, 0.2% FBS; *plain symbols*, 10%. PH group: *circle*, PAH; *triangle*, non PAH-PH. **(A, B) **or 5% **(C) **FBS. For each patient, 1 to 3 independent experiments were performed in triplicate. Regarding EC from the lung donor, 3 independent experiments were performed in triplicate.

In the presence of 10% FBS, CTEPH-PASMC mitogenic activity was significantly increased by 220% compared to PH-PASMC (48%) and to donor-PASMC (16%) (Figure 7A). In the presence of 10% FBS, CTEPH-PASMC migration capacity was significantly increased by 126% compared to PH-PASMC (54%) and to donor-PASMC (3%) (Figure 7B). In the presence of 5% FBS, CTEPH-PAEC mitogenic activity was significantly increased (by 120%) compared to PH-PAEC (26%) and to donor-PAEC (Figure 7C).

### Cellular composition of (sub)segmental pulmonary arteries in CTEPH patients

**Figure 8:**
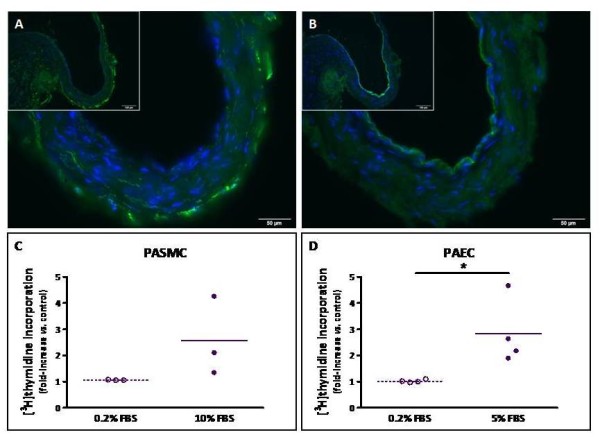
**Cellular composition and *in vitro *proliferative capacities of PAEC and PASMC from (sub)segmental pulmonary arteries of CTEPH patients**. Serial contiguous sections of (sub)segmental pulmonary arteries from CTEPH patients were stained with antibodies raised against α-SMA (A) or CD31 (B). Nuclei were counterstained using DAPI (blue). Magnification: main image, 40 × (bar, 50 μm); insert, 20 × (bar, 100 μm). Subconfluent cells were starved for 24 h in 0.2% FBS medium. Mitogenic activity was measured in the presence of 0.2% (control) or 10% FBS **(C) **or 5% FBS **(D) **as described above. (D) *p = 0.03.

Staining of a sub-segmental pulmonary artery with α-SMA (Figure 8A) and CD31 (Figure 8B) antibodies showed a mild neointima (Figure 8A) and an intact endothelium (Figure 8B). In the presence of 5 and 10% FBS, mitogenic activity of PAEC and PASMC isolated from segmental or sub-segmental pulmonary arteries of CTEPH patients was increased by 184% and by 156%, respectively (Figure 8C and 8D).

## Discussion

The aim of the present study was to characterize PASMC and PAEC isolated from large arteries of CTEPH patients and to investigate a potential role of both PASMC and PAEC in the vascular remodeling resulting in permanent obstruction of large pulmonary arteries in CTEPH patients. Our results reveal that, in CTEPH patients, smooth muscle differentiation markers were down-regulated in proximal pulmonary vascular tissue and that pulmonary vascular cells had enhanced proliferative and migratory capacities *in vitro*, suggesting a potential contribution of PASMC and PAEC to vascular remodeling in proximal pulmonary arteries in CTEPH.

The use of primary cell cultures implies a selection of cells with enhanced growth potential. However, both CTEPH and PH cells were isolated following the same protocol and all experiments were performed with cells at a low number of passages. PEA material usually consists of thrombotic and fibrotic material obstructing the main pulmonary arteries and 40% of the PEA material we have collected did not harbor any visible thrombotic material. However, the material used to establish primary cell culture was free of any thrombotic material and comprises a part of the media and a thickened neointima. Proximal pulmonary arteries isolated from non-thomboembolic PH patients and from lung donors comprise the whole media and a moderately thickened intima. Consequently, in all groups of patients PAEC and PASMC have been isolated from the vascular wall and not from the thrombus. Moreover, despite exposure to similarly high pressures in CTEPH and non-thromboembolic PH, pulmonary vascular cells from non-thromboembolic PH patients harbor similar proliferative and migratory properties as those from lung donors, who had no PH. Finally, while sub-segmental pulmonary arteries from CTEPH patients displayed only mild neointima thickening and intact endothelium by contrast to proximal arteries, both PASMC and PAEC harbored enhanced proliferative capacities similar to that observed in proximal pulmonary arteries.

### Pulmonary vascular cell characterization

Dysregulation of thrombosis and thrombolysis observed in some patients with CTEPH [5] is not sufficient to explain the intraluminal thrombus organization and fibrous vessel obliteration. Moreover, proximal pulmonary vessel remodeling may even occur without evidence of any clot formation [14]. Increased cellularity has been observed in proximal vascular lesions [8], but cellular protagonists have not been clearly identified. Few studies have focused on the implication of pulmonary artery cells in the pathogenesis of the disease. Lang and co-workers have investigated tissue plasminogen secretion by EC derived from main pulmonary arteries of patients with CTEPH [15]. Firth and Yao highlighted the presence of endothelial and multipotent mesenchymal progenitor cells in pulmonary vascular tissue of CTEPH patients [16,17]. Considering that the physiopathology of CTEPH remains incompletely understood, establishing well characterized primary cultures of proximal pulmonary vascular cells from CTEPH patients should be an asset to unravel the mechanisms resulting in the persistence of unresolved thrombi in CTEPH. PAEC express CD31 at their surface, vWF in Weibel-Palade bodies and are capable of taking up ac-LDL, confirming the homogeneity of our PAEC primary cultures, as described elsewhere [18]. Consequently, the combination of a mild enzymatic digestion coupled to purification on CD31 antibody-conjugated magnetic beads can be validated to establish homogeneous PAEC primary cultures. Despite of differences between neointimal material from CTEPH and non-thromboembolic patients, both primary cultures of PASMC harbor SM differentiation marker expression including α-SMA, desmin and SMMHC.

### PASMC proliferative phenotype

Regarding pulmonary arterial tissue content in smooth muscle differentiation markers, expression of desmin, a marker of late smooth muscle differentiation, was significantly lower in CTEPH than in PH. By contrast, α-SMA expression, a marker of early smooth muscle differentiation, was similar in both groups of patients, suggesting a potential switch of CTEPH-PASMC towards a proliferative phenotype. Moreover, both medial and PH-PASMC concomitantly express α-SMA and desmin or SMMHC, whereas neointimal CTEPH-PASMC mostly harbor α-SMA labeling. In addition, neointima thickness of the proximal pulmonary arteries was significantly higher in CTEPH than in non-thromboembolic patients. Taken together, these findings could suggest that CTEPH-SMC likely derived from clusters of PASMC with a switched proliferative phenotype. Accordingly, dedifferentiation or phenotypic modulation of SMC has been widely depicted in various vascular wall diseases including atherosclerosis [19]. This process is accompanied by a down-regulation of several smooth muscle differentiation markers including myosin heavy chain, α-actin, desmin, smoothelin, caldesmon and by an increase of their proliferating and migrating capacities *in vitro *[19]. In the present study, we have evidenced that neointima of proximal pulmonary arteries from CTEPH patients express less late SM differentiation markers and contain SMC with a proliferative phenotype, which display enhanced proliferating and migratory capacities *in vitro*. However, we cannot exclude that CTEPH-SMC may also derive from progenitor cells since Firth et al have identified a myofibroblast cell phenotype within the multipotent progenitor cell population isolated from CTEPH patients [16].

### Pulmonary vascular dysfunction

Our results indicate a potential involvement of the vascular wall in the pathophysiological process of CTEPH, as suggested by *in vitro *increased proliferating and migrating capacity of proximal CTEPH-PASMC and enhanced mitogenic activity of proximal CTEPH-PAEC. By contrast, enhanced *in vitro *proliferating capacity of distal precapillary PASMC and PAEC has been described in PAH [20,21]. Hyperproliferative apoptosis-resistant EC [22] and alterations of cellular bioenergetics of PAEC have been evidenced in idiopathic PAH [23]. Remodeling of the pulmonary vascular wall, characterized by intima and media hypertrophy, is a key process contributing to the formation of plexiform lesions observed in distal pulmonary vessels in PAH [24-26]. Endothelium dysfunction, accounted by hyper-proliferative CTEPH-PAEC, could contribute to PASMC migration and proliferation through PAEC protease-induced matrix impairment and PAEC-derived growth factors, respectively. As mentioned above, the hyperproliferative phenotype of both proximal and sub-segmental pulmonary vascular cells also suggest that these changes would not be only attributable to effects of thrombus-derived compounds, but also to cellular intrinsic modifications.

### Relevance of the study

To date, few studies had focused on the pathophysiology of CTEPH. By establishing primary cultures of PAEC and PASMC derived from CTEPH patients, we aim to unravel the mechanisms potentially involved in the persistent obstruction of proximal pulmonary arteries following massive or recurrent pulmonary embolism. It appears that enhanced proliferation and/or migration of PAEC and PASMC could be involved in the progression of the disease. Although our results are mainly based on primary cell culture bypassing intercellular communications, shear stress and dynamic effects of blood flow, it remains an interesting tool to further understand the pathophysiology of CTEPH.

## Conclusion

Modified proliferative and/or migratory responses of primary PASMC and PAEC *in vitro*, associated to a proliferative phenotype of CTEPH-PASMC suggest a dysfunction of pulmonary vascular cells in CTEPH, potentially involved in vascular remodeling.

## Abbreviations

α-SMA: α-smooth muscle actin; ac-LDL: acetylated-LDL; CTEPH: chronic thromboembolic pulmonary hypertension; FBS: fetal bovine serum; PAEC: pulmonary arterial endothelial cell; PAH: pulmonary arterial hypertension; PASMC: pulmonary arterial smooth muscle cell; PEA: pulmonary endarterectomy; SMMHC: smooth muscle myosin heavy chain; sPAP: systolic pulmonary arterial pressure; vWF: von Willebrand Factor.

## Competing interests

The authors declare that they have no competing interests.

## Authors' contributions

MW has acquired, analyzed and interpreted the data, performed statistical analysis and drafted the manuscript. RQ has conceived and designed the research, acquired, analyzed and interpreted the data, performed statistical, handled supervision and made critical revision of the manuscript. AR has acquired the data. MRS and FW have made critical revision of the manuscript. DVR and BM have collected the human tissue specimens and made critical revision of the manuscript. MD has conceived and designed the research, handled funding and supervision and made critical revision of the manuscript. All authors read and approved the final manuscript.

## Supplementary Material

Additional file 1 **Detailed Methods**. Click here for file

Additional file 2 **Table Individual patient characteristics**. Click here for file
