# Supplementary material for: Characterization of proximal pulmonary arterial cells from chronic thromboembolic pulmonary hypertension patients
Source: Respir Res. 2012 Mar 27;13(1):27. doi: 10.1186/1465-9921-13-27 (PMC3352254; doi:10.1186/1465-9921-13-27)
Supplement: Additional file 1 — Detailed Methods. [file 1465-9921-13-27-S1.PDF]

Characterization of proximal pulmonary artery cells in chronic thromboembolic pulmonary hypertension patients

Rozenn Quarck, Marijke Wynants, Alicja Ronisz, Maria Rosario Sepulveda, Frank Wuytack, Dirk Van Raemdonck, Bart Meyns, Marion Delcroix

METHODS

Isolation of PAEC and PASMC

Proximal PAEC were obtained by collagenase (1 mg.mL^{-1}) digestion in HBSS for 20 min at 37°C. PAEC were seeded on gelatin-coated cell culture flasks. Immunomagnetic isolation of PAEC was performed using anti-CD31 monoclonal antibody–labeled beads, according to the manufacturer's instructions (Miltenyi Biotec, Utrecht, The Netherlands). Proximal PASMC were isolated using an explant-outgrowth method [1]. PASMC were seeded on fibronectin-coated cell culture flasks. All experiments were carried out with cells which have undergone less than 7 passages.

Cell characterization

PAEC phenotype was characterized by labeling cells with Dil-Ac-LDL (Tebu-Bio, Le Perray en Yvelines, France) and by immunofluorescence using antibodies against CD31 (clone JC70A; Dako, Belgium) and against vWF (clone F8/86; Dako, Belgium). PASMC phenotype was characterized by immunocytochemistry using antibodies against α-SMA, (clone 1A4; Dako, Belgium), desmin (abcam, Cambridge, UK) and human SMMHC (Biomedical Technologies, Stoughton, MA). Desmin and SMMHC are markers of mature and differentiated SMC, whereas α-SMA is expressed at early stages of the differentiation of the smooth muscle [2].

Cells seeded onto gelatine-coated chamber slides (Lab-Tek, Nunc) were washed with phosphate-buffered saline (PBS) and fixed with 4% paraformaldehyde in PBS for 20 min at room temperature. The cells were permeabilized with 0.2% Triton-X100 in PBS, and blocked for 1 h with 3% bovine serum albumine in PBS. The localization of proteins was performed by using one or a mixture of two primary antibodies diluted in the blocking solution for 2 h (CD31, 1:25 dilution; vWF, 1:20

dilution; α-SMA, 1:100 dilution; desmin, 1:25 dilution; SMMHC, 1:50 dilution). Fluorescence labelling was obtained using the secondary antibodies Alexa488 goat anti-mouse, Alexa488 goat anti-rabbit at 1:2000 dilution (Invitrogen, Merelbeke, Belgium) for 1 h. Proliferating PAEC were incubated in the presence of 10µg/mL Dil-Ac-LDL for 4h at 37°C and fixed with 4% paraformaldehyde in PBS for 20 min at room temperature. Staining with 10 µM 4´,6-diamidino-2-phenylindole (DAPI) was used to visualize nuclei. FluorSave was used as mounting medium and slides were analyzed using an inverted Olympus IX81 fluorescence microscope. Negative controls were performed for every set of experiments by omitting the primary antibodies from the procedure.

Expression of PASMC markers in pulmonary vascular tissue by immunofluorescence

Localization of α-SMA, desmin and SMMHC has been performed on 7-µm tissue cryosections by immunofluorescence using antibodies against α-SMA, desmin and SMMHC.

Fresh tissue was fixed overnight in 4% paraformaldehyde in PBS, gelatin-embedded and frozen in liquid nitrogen. 7-µm tissue cryosections were performed. Tissue sections were permeabilized using 0.05% Triton-X100 in PBS and blocked for 1 h in PBS containing 0.25% Triton-X100, 2 $g.L^{-1}$ gelatin and 0.1 M lysine. The localization of proteins was performed overnight at room temperature by using a mixture of two primary antibodies diluted in PBS containing 0.25% Triton-X100 and 2 $g.L^{-1}$ gelatin (α-SMA, 1:100 dilution; desmin, 1:25 dilution; SMMHC, 1:50 dilution). Fluorescence labelling was obtained using a mixture of the secondary antibodies Alexa488 goat anti-mouse and Alexa594 goat anti-mouse (1:750 dilution) for 1 h. Staining with 10 µM 4´,6-diamidino-2-phenylindole (DAPI) was used to visualize nuclei. FluorSave was used as mounting medium and slides were analyzed using an inverted Olympus IX81 fluorescence microscope. Negative controls were performed for every set of experiments by omitting the primary antibodies from the procedure.

Western blotting

Pulmonary vascular tissue was homogenized in an ice-cold buffer containing 50mM Tris , pH 8.0, 10 mM CHAPS, 2 mM EDTA, 2 mM EGTA, 1µg/mL leupeptin and 1µg/mL antipain. Insoluble material was removed by centrifugation at 12,000 g and for 4°C for 5 min. Protein concentrations were determined using the BCA assay kit with bovine serum albumin as standard. Tissue and cell lysate proteins were solubilized in Laemmli buffer and reduced in the presence of β-mercaptoethanol for 5 min at 95°C and separated by 12% sodium dodecyl sulfate-polyacylamide gel electrophoresis (SDS-PAGE) prior to electroblotting on polyvinylidene fluoride (PVDF) filters. Filters were quenched overnight at 4°C by incubation in Tris-buffered saline (TBS: 10mM Tris, pH 7.5, 150mM NaCl) containing 0.1% Tween-20 (w/v) and 5% fat-free dry milk (w/v). The PVDF filters were incubated for 2 h at room temperature with the following antibodies: anti-αSMA (dilution, 1:10,000), anti-desmin (dilution, 1:1000) and β-actin (dilution, 1:5000; abcam, Cambridge, UK) in the quenching solution, washed in TBS containing 0.1% Tween-20 and incubated for 1 h at room temperature with horseradish peroxidase-conjugated donkey anti-rabbit IgG (dilution 1:10,000) for anti-desmin and anti-mouse IgG (dilution 1:10,000) for anti-αSMA and anti-βactin. Peroxidase staining was revealed with a chemiluminescence kit (GE Healthcare, Chalfont St. Giles, UK) and performed with films exposed at room temperature. Protein expression was quantified by densitometric measurement of the chemiluminscent bands the Photoprint imaging system (Vilber Lourmat) coupled to the software (Vilber Lourmat).

Cell migration scratch wound assay

Cell migration has been evaluated using a scratch wound assay [1]. The cell monolayer was scratched with a rubber policeman to generate a wound with a width of approximately 10 mm. The remaining cells were washed twice with 0.2% FBS and 0.2% or 10% FBS containing medium was added for 36 hours. PASMC were fixed in methanol, nuclei were stained using the May-Grünwald-Giemsa method and the number of cells that have migrated over the scratch wound limit was assessed in 10 high power fields along the scrape line.

REFERENCES

1 Theilmeier G, Quarck R, Verhamme P, Bochaton-Piallat ML, Lox M, Bernar H, Janssens S, Kockx M, Gabbiani G, Collen D, Holvoet P: **Hypercholesterolemia impairs vascular remodelling after porcine coronary angioplasty**. *Cardiovasc Res* 2002, **55**:385-395.

2 Owens GK: **Regulation of differentiation of vascular smooth muscle cells**. *Physiol Rev* 1995, **75**:487-517.
