# Supplementary material for: Characterization of proximal pulmonary arterial cells from chronic thromboembolic pulmonary hypertension patients
Source: Respir Res. 2012 Mar 27;13(1):27. doi: 10.1186/1465-9921-13-27 (PMC3352254; doi:10.1186/1465-9921-13-27)
Supplement: Additional file 2 — Table Individual patient characteristics. [file 1465-9921-13-27-S2.PDF]

Characterization of proximal pulmonary artery cells in chronic thromboembolic pulmonary hypertension patients

Rozenn Quarck, Marijke Wynants, Alicja Ronisz, Maria Rosario Sepulveda, Frank Wuytack, Dirk Van Raemdonck, Bart Meyns, Marion Delcroix

Table. Individual patient characteristics

**NON-THROMBOEMBOLIC**							
**Patient**	**Cause of PH**	**Age** *(years)*	**Gender**	**mPAP** *(mm Hg)*	**RAP** *(mm Hg)*	**CO** *(L.min^{-1})*	**TPR** *(dyne.sec.cm^{-5})*
1	IPAH	27	F	55	13	2,9	1733
2	IPAH	56	M	*(64)*	*(15)*	*(5.6)*	*(923)*
3	IPAH	42	M	30	NA	9,2	264
4	EAA	44	M	33	22	6,2	422
5	COPD	59	M	30	15	7,2	330
6	sarcoidosis	44	M	48	11	NA	NA
7	IPAH	41	F	*(60)*	*(13)*	*(3.2)*	*(1481)*
8	IPAH	37	F	45	14	5,4	662
9	IPF	64	M	51	8	5,1	805
10	EAA	52	M	43	11	4,5	764
11	PAH-CHD	45	V	*(71)*	*(14)*	*(2.4)*	*(2328)*
12	bronchiectasis	61	M	37	11	6,3	470
**CTEPH**							
**Patient**		**Age** *(years)*	**Gender**	**mPAP** *(mm Hg)*	**RAP** *(mm Hg)*	**CO** *(L.min^{-1})*	**TPR** *(dyne.sec.cm^{-5})*
1		47	F	62	17	3,00	1662
2		71	F	65	14	1,70	3043
3		69	M	37	10	2,40	1233
4		59	F	40	20	2,2	1455
5		77	F	34	9	1,80	1526
6		59	F	47	13	3,20	1167
7		77	F	33	8	3,00	871
8		44	M	41	15	4,30	769
9		45	F	59	18	4,60	1020
10		38	F	23	14	3,3	558
11		56	F	31	8	2,60	954
12		55	F	50	3	3,10	1282
13		77	M	30	7	2,60	923
14		75	F	46	8	3,2	1150
15		44	F	49	9	5,3	740
16		53	F	30	9	2,87	936

mPAP, mean pulmonary arterial pressure; RAP, right atrial pressure; TPR, total pulmonary vascular resistance; CO, cardiac output. Hemodynamic parameters have been measured at the time of PEA or lung transplantationHemodynamic parameters measured at the time of PEA or lung transplantation or, if missing, at the last catheterization preceding lung transplantation *(in italic between brackets)*. COPD, chronic obstructive pulmonary disease; EAA, extrinsic allergic alveolitis; IPAH, idiopathic pulmonary arterial hypertension; IPF, idiopathic pulmonary fibrosis; PAH-CHD, pulmonary arterial hypertension associated with congenital heart disease
